# Specific Substrate Activity of Lotus Root Polyphenol Oxidase: Insights from Gaussian-Accelerated Molecular Dynamics and Markov State Models

**DOI:** 10.3390/ijms251810074

**Published:** 2024-09-19

**Authors:** Minghao Liu, Siyun Zheng, Yijia Tang, Weiwei Han, Wannan Li, Tao Li

**Affiliations:** 1 Key Laboratory for Molecular Enzymology and Engineering of Ministry of Education, School of Life Sciences, Jilin University, Changchun 130012, China; lmh23@mails.jlu.edu.cn (M.L.); weiweihan@jlu.edu.cn (W.H.); 2 School of Life Sciences, Jilin University, Changchun 130012, China; zhengsy1322@mails.jlu.edu.cn (S.Z.); tangyijia123456@mails.jlu.edu.cn (Y.T.); liwannan@jlu.edu.cn (W.L.)

**Keywords:** polyphenol oxidase, conformational changes, molecular dynamics simulation, Markov state model

## Abstract

Polyphenol oxidase (PPO) plays a key role in the enzymatic browning process, and this study employed Gaussian-accelerated molecular dynamics (GaMD) simulations to investigate the catalytic efficiency mechanisms of lotus root PPO with different substrates, including catechin, epicatechin, and chlorogenic acid, as well as the inhibitor oxalic acid. Key findings reveal significant conformational changes in PPO that correlate with its enzymatic activity. Upon substrate binding, the alpha-helix in the Q53-D63 region near the copper ion extends, likely stabilizing the active site and enhancing catalysis. In contrast, this helix is disrupted in the presence of the inhibitor, resulting in a decrease in enzymatic efficiency. Additionally, the F350-V378 region, which covers the substrate-binding site, forms an alpha-helix upon substrate binding, further stabilizing the substrate and promoting catalytic function. However, this alpha-helix does not form when the inhibitor is bound, destabilizing the binding site and contributing to inhibition. These findings offer new insights into the substrate-specific and inhibitor-induced structural dynamics of lotus root PPO, providing valuable information for enhancing food processing and preservation techniques.

## 1. Introduction

Polyphenol oxidases (PPOs) are a class of copper-containing enzymes that are widely distributed in plants, fungi, bacteria, and animals [1]. These enzymes play a key role in the enzymatic browning process [2], which results in the formation of brown pigments when phenolic compounds in fruits [3] and vegetables [4] are oxidized to quinones. This browning reaction can significantly affect the nutritional quality, visual appeal, and commercial value of food products [5]. In recent years, the study of PPOs has received much attention due to their dual role in the browning reaction [6,7,8]. On the one hand, PPO activity is essential for the development of flavor, color, and antioxidant properties of certain foods such as tea [9], coffee [10], and cocoa [11]. On the other hand, enzymatic browning of fresh produce such as apples [12], potatoes [13], and avocados [14] poses a major challenge to the food industry, leading to considerable economic losses and waste.

The molecular mechanisms of PPO activity, substrate specificity, and inhibition have been extensively studied, and many studies have been devoted to elucidating the structure–function relationships of these enzymes [15]. In recent years, advances in computational and experimental techniques have provided deeper insights into the catalytic mechanisms of PPOs [16]. For example, molecular docking and molecular dynamics (MD) simulations have become powerful tools for studying the binding interactions between PPOs and various substrates or inhibitors [17]. These studies have revealed key information about active pocket, substrate-binding modes, and the dynamic behavior of PPOs during catalysis.

In addition, identifying and characterizing natural and synthetic PPO inhibitors have opened new avenues for controlling enzymatic browning in foods. Natural inhibitors such as polyphenols, peptides, and small organic molecules have shown promising potential for reducing PPO activity and extending the shelf life of fresh produce [18]. Recent studies have also explored genetic engineering approaches to down-regulate PPO expression in transgenic plants to mitigate browning and improve produce quality [19].

Lotus root (*Nelumbo nucifera*) is a specific source of PPOs and is of interest for its abundance of bioactive phytochemicals such as polyphenols and flavonoids. Lotus root is known for its numerous health benefits, such as antioxidant, anti-inflammatory, and anti-cancer properties [20]. However, like other fresh produce, lotus root is susceptible to enzymatic browning after cutting, which affects its color, flavor, and nutritional value. The main cause of browning is the PPO-catalyzed oxidation of polyphenols to produce quinones, which subsequently polymerize to form brown pigments [21].

It was reported that the mechanism of the specific selection of polyphenol substrates by polyphenol oxidase (PPO) in lotus roots reveals the biochemical basis of browning in lotus roots. After identification and purification of lotus root PPO, it was found to have the highest catalytic activity at 35 °C and pH 6.5, and its catalytic activity towards a wide range of substrates was identified [22]. Despite these advances, there are still gaps in our understanding of the specific molecular interactions and catalytic mechanisms between PPO extracted from lotus roots and various substrates. This present study aims to fill this knowledge gap by exploring the reaction mechanisms of PPO from lotus root through Gaussian-accelerated molecular dynamics (GaMD) simulation methods. We specifically selected three substrates from a previously studied set of five, focusing on those with the first, third, and fifth highest catalytic activities—(+)-catechin, (−)-epicatechin, and chlorogenic acid, respectively [22]. This selection was made to provide a clearer differentiation of substrate specificity, allowing us to better elucidate the unique catalytic behavior of lotus root PPO. Catechin and epicatechin, both flavonoids, possess multiple hydroxyl groups on their aromatic rings, which enhance their solubility in polar solvents and contribute to their strong antioxidative properties. These hydroxyl groups also facilitate participation in acid–base reactions and electrophilic substitution, making them ideal candidates for studying PPO’s substrate interactions. Chlorogenic acid, a polyphenol ester, contains polar functional groups that participate in electrophilic and nucleophilic reactions, and its hydroxyl groups further contribute to its electron-donating capacity and antioxidative properties. To further investigate the inhibitory mechanisms of lotus root PPO, we included oxalic acid, a well-established inhibitor from a previous study [22], to assess its influence on substrate specificity and better understand its effects on the enzyme’s activity. Oxalic acid, a small dicarboxylic acid, lacks the aromatic structures seen in the other substrates but exhibits distinct behavior due to its highly polar carboxyl groups, which allow it to chelate metal ions and interfere with PPO’s catalytic function. GaMD makes the potential energy surface smoother by adding a harmonic lifting potential to the potential energy surface of the system, which significantly reduces the energy barriers and improves the sampling efficiency [23]. GaMD significantly enhances the sampling ability of the system, enabling the capture of more comprehensive conformational changes and kinetic processes [24], which helps to reveal the details of the catalytic mechanism of PPO. In this study, 500 ns Gaussian-accelerated molecular dynamics (GaMD) simulations were performed for five systems: ligand-free protein, catechin-bound protein, epicatechin-bound protein, chlorogenic acid-bound protein, and oxalic acid-bound protein as inhibitors. The simulations revealed that the alpha-helix in the Q53-D63 region, located near the copper ion, extended during substrate binding, likely stabilizing the active site and enhancing catalytic efficiency. However, in the presence of the inhibitor, this helix was disrupted, reducing enzymatic activity. Additionally, a new alpha-helix was observed in the F350-V378 region at the substrate-binding site upon substrate binding, which may have further stabilized substrate interactions. Markov state model-based flux analysis and MM-PBSA energy analysis were also performed to further investigate the catalytic mechanisms. Detailed molecular dynamics simulations and energy calculations were performed to further understand the specificity of PPO for different substrates.

The understanding of these mechanisms in this study has deepened our knowledge of PPO function and substrate selectivity, providing a theoretical basis for optimizing food processing and storage methods to improve the preservation and quality of lotus root and other PPO-rich foods. This study also revealed the molecular basis of substrate specificity, which can help design more effective inhibitors or modifications of enzyme activity. Understanding the catalytic efficiency of PPO on different substrates can help develop effective inhibitors or modulation techniques that can improve food quality, extend shelf life, and reduce spoilage with significant economic and environmental benefits. In addition, this study provides valuable insights into the role of PPO in plant biology, which will benefit plant growth, development, and disease resistance studies.

## 2. Results

### 2.1. Molecular Docking Results

**Figure 1:**
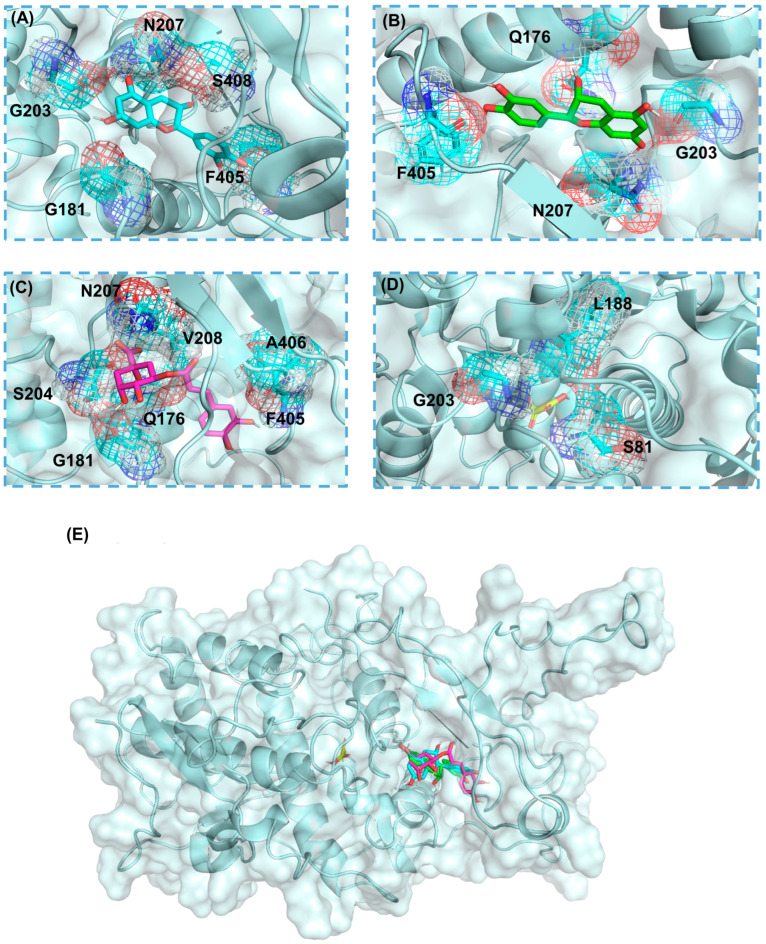
Binding pockets and interaction residues of polyphenol oxidase for (**A**) catechin, (**B**) epicatechin, (**C**) chlorogenic acid, and (**D**) oxalic acid. (**E**) Binding positions of four small molecules on polyphenol oxidase. Blue molecule represents catechin, green molecule represents epicatechin, pink molecule represents chlorogenic acid, and yellow molecule represents oxalic acid. The red color in the structure of the small molecule indicates an oxygen atom.

The molecular docking results for the interactions between the protein and the four compounds, as shown in Figure 1 and the corresponding supplementary figures, highlight crucial hydrogen bonds that stabilize the binding of each molecule within the protein’s active site. For (+)-catechin (Figure 1A and Figure S1), hydrogen bonds were formed with G181, G203, N207, F405, and S408, playing a key role in securing its position. (−)-Epicatechin (Figure 1B and Figure S2) similarly formed hydrogen bonds with N207, G203, Q176, and F405, ensuring stable interaction within the binding pocket. In the case of chlorogenic acid (Figure 1C and Figure S3), multiple hydrogen bonds were observed with residues G181, G203, N207, Q176, S204, V208, and F405, anchoring it effectively in the active site. Lastly, for oxalic acid (Figure 1D and Figure S4), hydrogen bonds were established with S81, L188, and G203, highlighting its strong inhibitory interaction with the protein. These hydrogen bonds are critical for the stability and proper function of each molecule in relation to the protein. The molecular docking affinities for the four compounds were as follows: catechin (−7.9 kcal/mol), epicatechin (−7.9 kcal/mol), chlorogenic acid (−8.1 kcal/mol), and oxalic acid (−4.0 kcal/mol).

### 2.2. Structural Stability and Flexibility between Substrates and PPO

**Figure 2:**
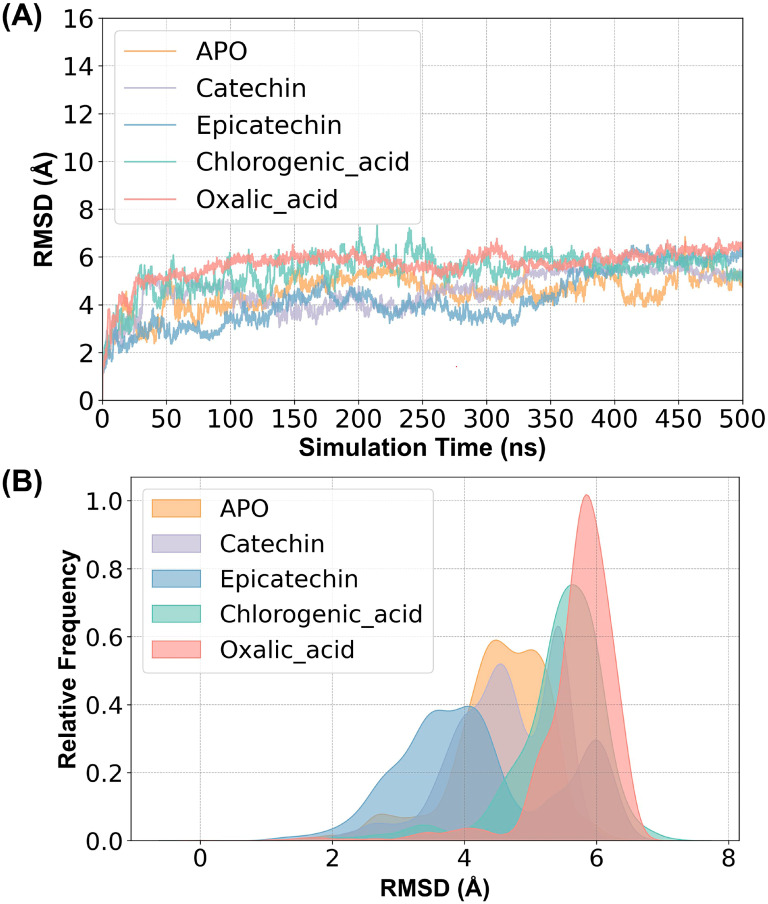
(**A**) RMSD values of the five simulated systems: ligand-free (APO), catechin-bound (catechin), epicatechin-bound (epicatechin), chlorogenic acid-bound (chlorogenic_acid) proteins, and oxalic acid-bound proteins (oxalic_acid); (**B**) RMSD kernel density distributions for the four simulated systems.

In this study, five molecular dynamics simulation systems were constructed, which are: ligand-free, catechin-bound, epicatechin-bound, chlorogenic acid-bound proteins, and oxalic acid-bound proteins, to investigate the effect of different substrate binding on the structure of PPO. The root mean square deviation (RMSD) analysis was performed for the four systems, as shown in Figure 2A. Their kernel density distribution curves are shown in Figure 2B. Calculation of the root mean square deviation (RMSD) of the Cα atom of the protein backbone allowed for assessment of the stability of the protein conformation [25]. The RMSD values for all five systems stabilized around 5 Å after fluctuation. Since the protein structure was not a resolved crystal structure but was predicted, the system was considered to be in equilibrium and could be used for further analysis.

**Figure 3:**
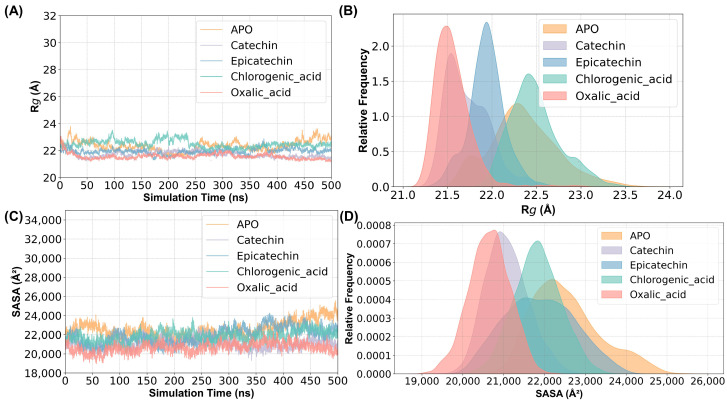
(**A**) R_g_ values of the five simulated systems: ligand-free (APO), catechin-bound (catechin), epicatechin-bound (epicatechin), chlorogenic acid-bound (chlorogenic_acid) proteins, and oxalic acid-bound (oxalic_acid) proteins. (**B**) R_g_ kernel density distributions for the five simulated systems. (**C**) SASA values of the five simulated systems. (**D**) SASA kernel density distributions for the five simulated systems.

The radius of gyration (R_g_) can reflect the plasticity potential of protein structures. As shown in Figure 3A,B, the R_g_ values of the oxalic acid system, fluctuating between 21.00 and 21.75 Å, indicate that oxalic acid, as an inhibitor, made the protein structure more compact and stable, likely suppressing enzyme activity. In contrast, the catechin and epicatechin systems, with R_g_ values fluctuating between 21.50 and 23.50 Å, suggest that these substrates increased the fluffiness of the protein structure. The chlorogenic acid system showed R_g_ values between 21.00 and 22.50 Å, indicating a more compact structure compared to catechin and epicatechin, which aligns with its lower catalytic activity [26]. The solvent-accessible surface area (SASA) value responds to the area of the surface of a protein or protein–ligand complex exposed to the solvent [27]. As shown in Figure 3C,D, the oxalic acid system has the lowest SASA values, indicating that inhibitor binding results in the most compact and stable protein structure, reducing enzyme activity by limiting active site accessibility. The catechin system shows slightly higher SASA values than oxalic acid, with a modest increase in surface exposure but still relatively compact. Epicatechin and chlorogenic acid have similar, slightly higher SASA values compared to catechin, reflecting more surface exposure and a less compact structure. While increased SASA may improve active site accessibility and potentially enhance enzyme activity, excessive exposure could destabilize the protein and reduce catalytic efficiency.

### 2.3. Analysis of Conformational Changes

**Figure 4:**
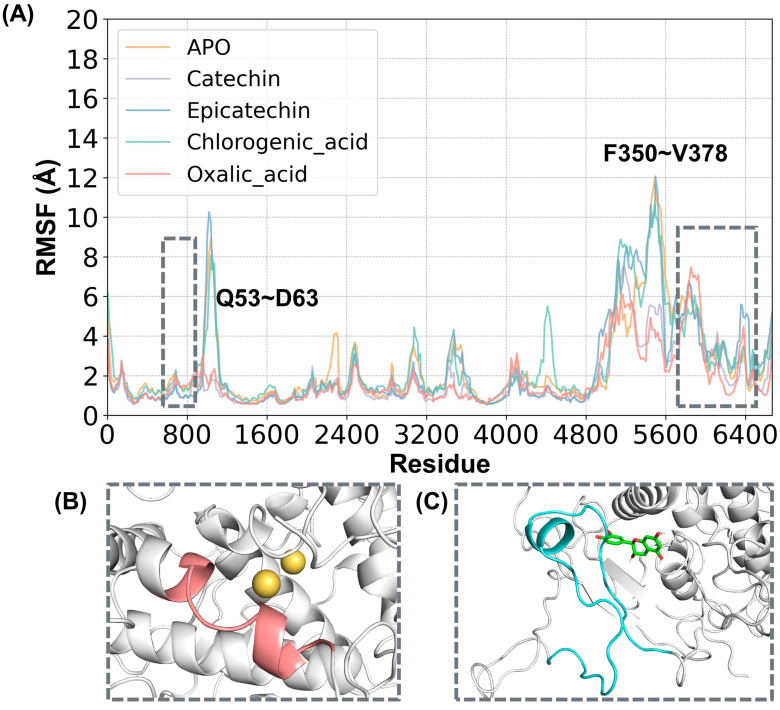
(**A**) RMSF values for the five simulated systems: ligand-free (APO), catechin-bound (catechin), epicatechin-bound (epicatechin), chlorogenic acid-bound (chlorogenic_acid) proteins, and oxalic acid-bound (oxalic_acid) proteins. (**B**) Secondary structure of the Q53-D63 region. (**C**) Secondary structure of the F350-V378 region. “Residue” represents the atom numbers of the amino acids of PPO. The yellow color indicates copper ions. Green molecules indicate substrate molecules.

In order to measure the atomic motions during kinetic simulations to reflect the flexibility of protein regional motions, root mean square fluctuation (RMSF) calculations were performed in this study. Figure 4A illustrates the RMSF values for the four systems. The labeled regions are Q53-D63 (shown in Figure 4B) and F350-V378 (shown in Figure 4C). Residues Q53-D63 are alpha-helical structures near the two copper ion positions. Residues F350-V378 are adjacent to the active pocket and cover the substrate-binding site. For these two residue regions, we performed further secondary structure change probability analysis.

**Figure 5:**
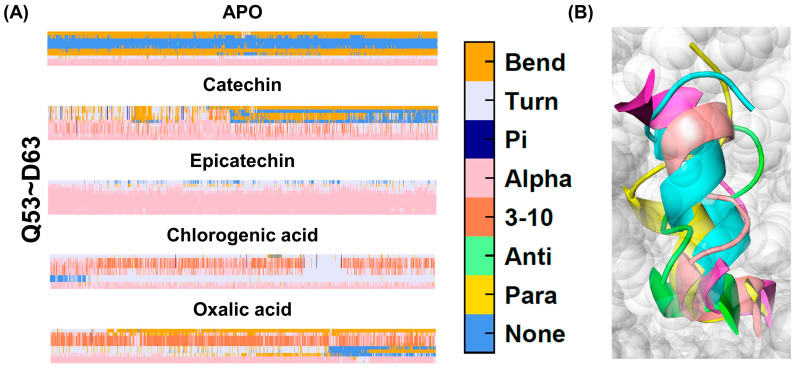
(**A**) The secondary structure timeline in the Q53-D63 region. (**B**) The secondary structure of the Q53-D63 region. Green represents the oxalic acid system, yellow represents the catechin system, blue represents the epicatechin system, purple represents the chlorogenic acid system, and pink represents the apo system.

The probability of secondary structure change at positions Q53-D63 is shown in Figure 5. We chose the 2500 frame of the 500 ns simulation trajectory for the schematic. Green represents the oxalic acid system, yellow represents the catechin system, blue represents the epicatechin system, purple represents the chlorogenic acid system, and pink represents the apo system. In both the apo and oxalic acid systems, the alpha-helix in this region unfolds. The copper center is a crucial structural domain in polyphenol oxidase (PPO), as it is directly involved in the enzyme’s catalytic activity [1]. This region is located in close proximity to the copper ion in polyphenol oxidase, which plays a crucial role in the enzyme’s catalytic activity. The unfolding of the alpha-helix near the copper ion likely disrupts the stability and positioning of the active site, reducing catalytic efficiency. Since oxalic acid is an inhibitor, its binding may induce or stabilize this structural disruption, thereby inhibiting enzyme activity by preventing proper substrate interaction with the copper ion.

**Figure 6:**
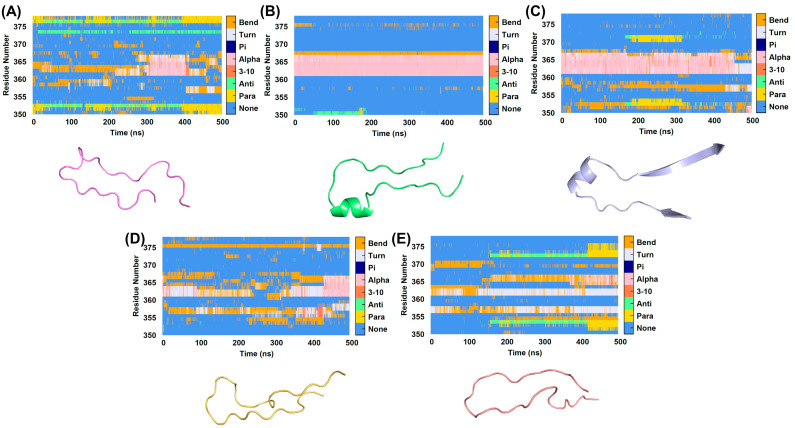
The secondary structure timeline in the F350-V378 region and the corresponding secondary structure diagrams for five systems: (**A**) ligand-free, (**B**) catechin-bound, (**C**) epicatechin-bound, (**D**) chlorogenic acid-bound proteins, and (**E**) oxalic acid-bound proteins.

The probability of secondary structure change at positions F350-V378 is shown in Figure 6. Residues covering the substrate-binding site play a crucial regulatory role in enzyme activity, as modifications in these regions can significantly enhance or inhibit catalytic efficiency by altering the enzyme’s interaction with the substrate [28]. This region, which lies above the substrate-binding site, is mainly composed of random coils and exhibits high flexibility. Upon substrate binding, particularly with catechin and epicatechin, an alpha-helix forms during the simulation, potentially stabilizing the substrate interaction and enhancing enzyme activity. In contrast, the alpha-helix in the apo system exists only briefly, suggesting reduced structural stability without a bound substrate. When the inhibitor oxalic acid is bound, the formation of the alpha-helix in this region is suppressed, which likely contributes to the inhibition of enzyme activity by preventing the structural stabilization needed for effective substrate binding and catalysis.

**Figure 7:**
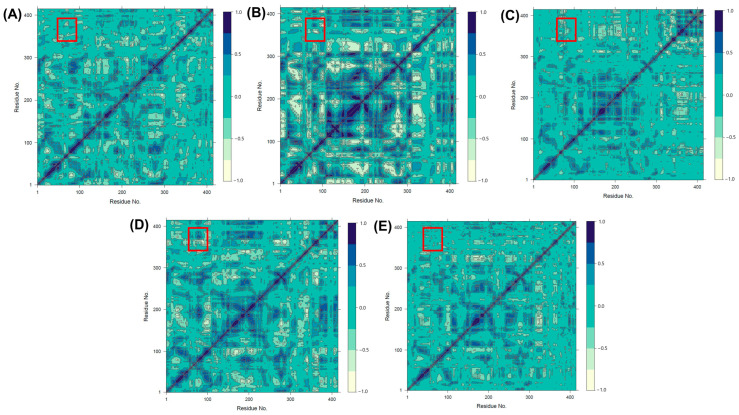
The Dynamic Cross-Correlation Matrix diagrams for five systems: (**A**) ligand-free, (**B**) catechin-bound, (**C**) epicatechin-bound, (**D**) chlorogenic acid-bound proteins, and (**E**) oxalic acid-bound proteins. Dark blue indicates positive correlations while light green indicates negative correlations. The red box highlights the interaction between Q53-D63 and F350-V378.

We used GaMD trajectories at equilibrium to calculate the inter-correlations between residues to explore the internal dynamics of PPO. Figure 7A–E shows the interaction correlation plots of the intermolecular motions associated between the distal regions of the proteins in the different complexes. The Dynamic Cross-Correlation Matrix (DCCM) in protein molecular dynamics simulations is used to identify and quantify the correlated movements between atoms or residues over time, helping to reveal functional relationships and allosteric interactions within the protein structure, which are critical for understanding enzyme mechanisms and structural dynamics [29]. After binding the substrates, the proteins of each system showed stronger interactions. Additionally, we observe a negative interaction between residues F350-V378 and those near Q53-D63, highlighted by red circles. The binding of oxalic acid disrupts this interaction. Structurally, the F350-V378 region, located above the substrate-binding site, and the Q53-D63 region, near the copper ion, play key roles in maintaining the enzyme’s functional conformation. The negative interaction between these regions may help regulate the flexibility and dynamics necessary for efficient catalysis. The disruption of this interaction by oxalic acid binding likely destabilizes this coordination, further inhibiting enzyme activity by preventing the proper structural alignment required for effective substrate processing and catalysis.

**Figure 8:**
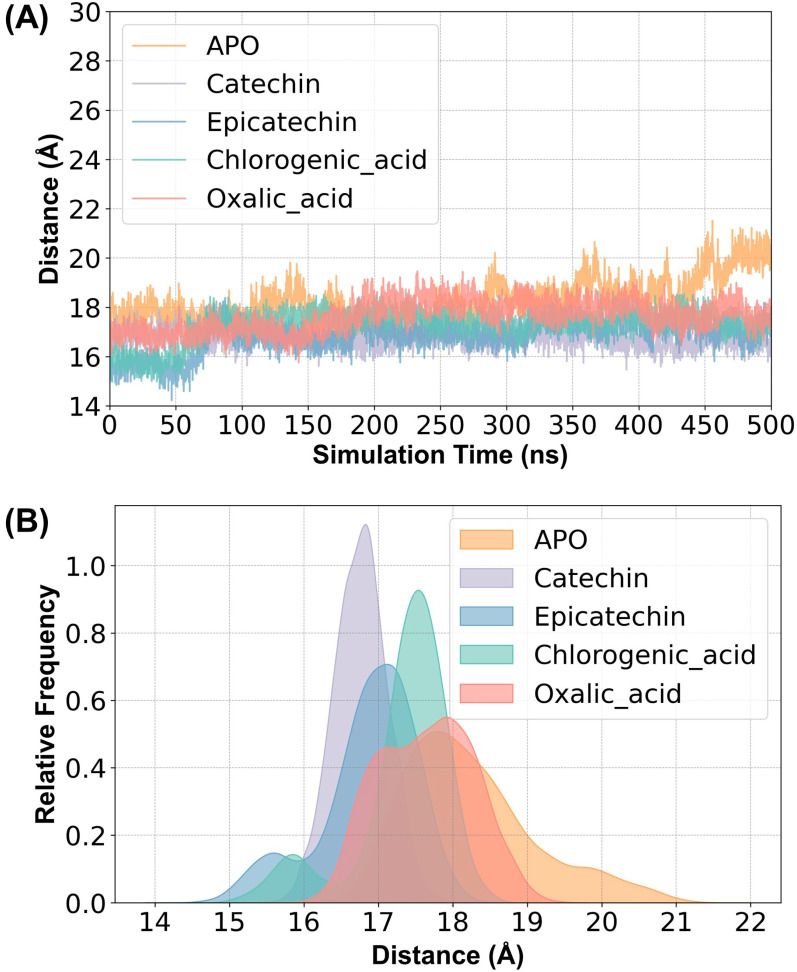
(**A**) Distance between the copper-coordinating histidines H88 and H214 in the five simulated systems: ligand-free (APO), catechin-bound (catechin), epicatechin-bound (epicatechin), chlorogenic acid-bound proteins (chlorogenic_acid), and oxalic acid-bound proteins (oxalic_acid). (**B**) Distance kernel density distributions for the five simulated systems.

The distance between the two histidine residues (H88 and H214) coordinating the copper ion in the enzyme is shown in Figure 8. The amino acids that coordinate copper ions in polyphenol oxidase (PPO), particularly histidine residues, are crucial for facilitating electron transfer during catalysis, which directly impacts the enzyme’s ability to oxidize phenolic substrates and regulate enzymatic browning reactions [30]. After substrate binding, these two residues move closer together, and the degree of this proximity correlates positively with the experimentally measured catalytic activity, indicating that a more compact coordination around the copper ion enhances enzyme function. In contrast, upon inhibitor binding, there is a slight increase in the distance between these residues, suggesting a disruption in the optimal copper ion coordination, which may lead to a reduction in catalytic efficiency. This highlights the importance of maintaining proper histidine coordination for effective enzyme activity. Previous studies have shown that a shorter distance between copper ions and histidine residues enhances PPO catalytic activity [16]. In comparison, our study similarly found that the reduced spacing between histidines coordinating the copper ion promotes increased PPO catalytic efficiency.

**Figure 9:**
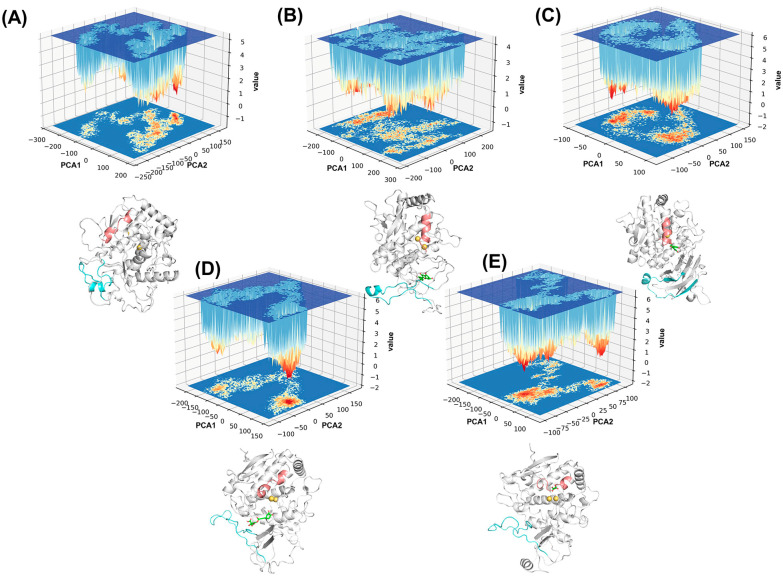
Free energy landscapes of the five systems: (**A**) ligand-free, (**B**) catechin-bound, (**C**) epicatechin-bound, (**D**) chlorogenic acid-bound proteins, and (**E**) oxalic acid-bound proteins. The lowest energy conformations are shown, highlighting the secondary structures of the Q53-D63 region (pink) and the F350-V378 region (cyan). The yellow color indicates copper ions. Green molecules indicate substrate molecules. The colors in the free energy landscape represent the energy levels, with red indicating lower free energy and blue indicating higher free energy.

The major conformational changes of the protein were determined with PCA analysis of the Cα atoms in the GaMD trajectory. The two largest eigenvalues, PCA1 and PCA2, were used as reaction coordinates to calculate the relative Gibbs free energies and to generate Gibbs free energy landscapes (FELs). The FELs provide valuable information about the different conformational states and reveal the energetic barriers of the proteins between conformations or states [31]. Figure 9 illustrates the free energy surface maps of the five systems with the lowest energy conformations, with different structural trends in the two main active regions identified by the previous analysis.

In Figure 9A, the free energy surface diagram of the ligand-free protein system is shown. After comparative calculations, the global energy minimum (PCA1: 19.82, PCA2: 128.53) was chosen for the analysis. The corresponding conformation occurs at 197.3 ns. The alpha-helix in the Q53-D63 region unfolds, while a transient alpha-helix forms briefly in the F350-V378 residue region.

The catechin-bound system’s free energy surface diagram is displayed in Figure 9B. The global energy minimum (PCA1: −160.55, PCA2: 65.42) was selected for examination following comparison computations. At 354.80 ns, the corresponding conformation takes place.

The epicatechin system’s free energy surface diagram is shown in Figure 9C. The global energy minima (PCA1: −60.62, PCA2: −54.39) were selected for examination following comparison computations. At 3.30 ns, the appropriate conformation takes place. For these two highly active substrates, the Q53-D63 region forms a relatively long alpha-helix, and an alpha-helix also develops in the F350-V378 residue region. In Figure 9D, the free energy surface diagram of the chlorogenic acid system is shown. After comparative calculations, the global energy minima (PCA1: 97.18, PCA2: −10.68) was chosen as the reference for the analysis. The corresponding conformation occurs at 313.00 ns. In the chlorogenic acid system, the alpha-helix in the Q53-D63 residue region shortens to some extent. Consistent with the secondary structure timeline analysis, the duration of the alpha-helix in the F350-V378 residue region is also reduced, presenting as a random coil in the principal component structure diagram.

The free energy surface diagram of the oxalic acid system is displayed in Figure 9E. The global energy minimum (PCA1: −14.45, PCA2: −51.52) was identified after performing comparison calculations. The corresponding conformation occurs at 238.20 ns. Upon inhibitor binding, the alpha-helix in the Q53-D63 residue region significantly shortens, and the F350-V378 residues remain in a random coil structure. The PCA analysis results are consistent with the earlier secondary structure analysis, supporting the regulatory role of these two regions in the catalytic activity of polyphenol oxidase.

To further investigate the relative steady states of each system and the key secondary structure changes during the simulation, we performed a Markov state model analysis of the trajectories.

**Figure 10:**
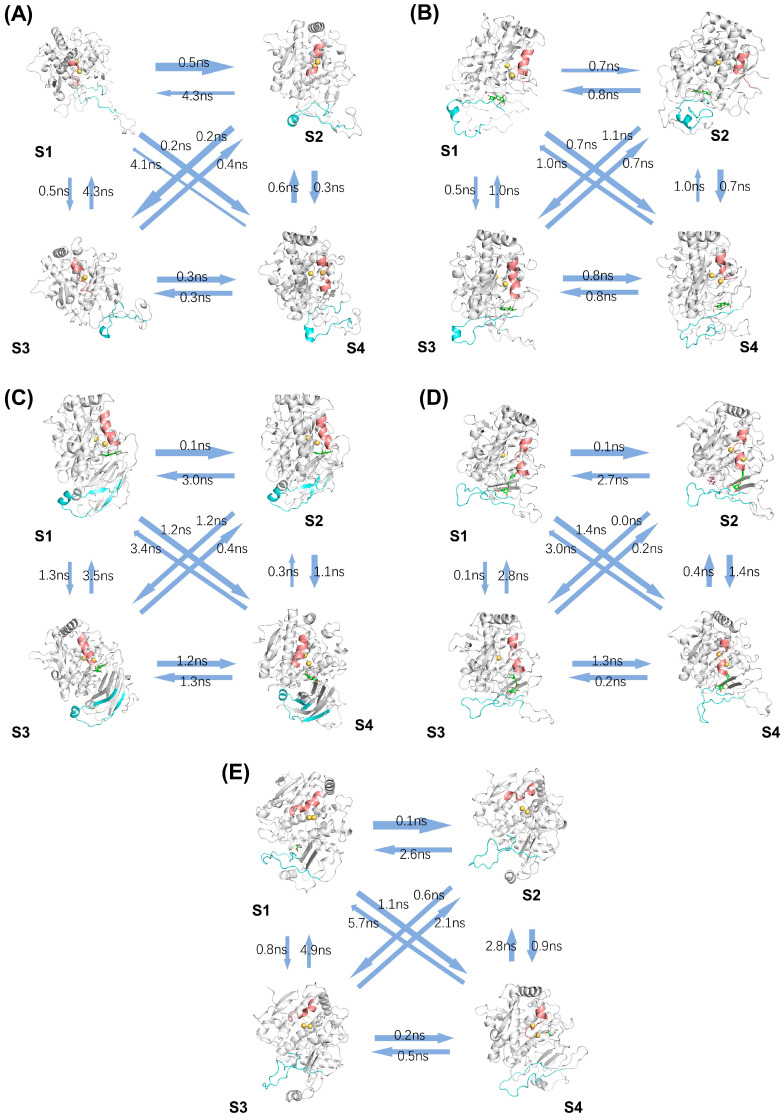
Flux analysis of the five systems: (**A**) ligand-free, (**B**) catechin-bound, (**C**) epicatechin-bound, (**D**) chlorogenic acid-bound proteins, and (**E**) oxalic acid-bound proteins, highlighting the secondary structures of the Q53-D63 region (pink) and the F350-V378 region (cyan). S1, S2, S3, and S4 represent the most significant metastable states identified in the Markov state model (MSM) analysis. The yellow color indicates copper ions. Green molecules indicate substrate molecules.

These states correspond to distinct conformational ensembles, and the flux between them illustrates the probability of transitions during the simulation. In the ligand-free system, as shown in Figure 10A, S3 and S4 represent more stable conformations. The Q53-D63 region consistently adopts a disrupted alpha-helix, while F350-V378 fluctuates between random coil and alpha-helix structures. In the catechin-bound system (Figure 10B), S3 and S4 represent stable states, with the Q53-D63 region maintaining a fully formed alpha-helix throughout. In the epicatechin-bound system (Figure 10C), S2 and S3 are stable conformations. In this system, Q53-D63 retains a stable long alpha-helix, and the F350-V378 region forms beta-sheets at the terminal regions while maintaining an alpha-helix in the central part, stabilizing the substrate binding and catalytic function. In the chlorogenic acid-bound system (Figure 10D), the Q53-D63 alpha-helix undergoes significant unwinding and flux analysis identifies S2 and S3 as stable conformations. The Q53-D63 alpha-helix shortens, and this destabilization likely reduces catalytic efficiency, aligning with chlorogenic acid’s lower activity observed experimentally. In the oxalic acid-bound system (Figure 10E), S3 and S4 represent stable conformations. Upon oxalic acid binding, the Q53-D63 region’s alpha-helix shortens significantly, while the F350-V378 region experiences helix unwinding and remains in a random coil state, further inhibiting enzymatic activity.

We also conducted free energy barrier calculations to quantify the transitions between these metastable states. The calculation of these barriers is crucial for understanding the kinetic pathways and timescales of the transitions, allowing us to better interpret how the system moves between different stable conformations [32]. The results of these calculations are presented in Table 1. These energy barriers provide further insight into the dynamics of the system, supporting the observed stability and conformational transitions between states.

### 2.4. MM-PBSA Analysis

The results of MM-PBSA calculations for the three systems—catechin, epicatechin, and chlorogenic acid—are summarized in Table 2. For catechin, the total binding free energy (ΔG_total_) is −11.53 ± 1.00 kJ/mol. For epicatechin, the ΔG_total_ is −10.75 ± 1.29 kJ/mol. For chlorogenic acid, the ΔG_total_ is −28.03 ± 1.04 kJ/mol. For oxalic acid, the ΔG_total_ is −0.31 ± 0.36 kJ/mol. These results indicate that among the three systems, chlorogenic acid shows the most favorable binding free energy, followed by catechin, epicatechin, and oxalic acid. In our molecular docking analyses, we found that more residues do interact with chlorogenic acid. This may explain the main reason why its G value is greater than that of the other substrates.

## 3. Discussion

This study provides valuable insights into the catalytic efficiency mechanisms of lotus root polyphenol oxidase (PPO) with various substrates—catechin, epicatechin, and chlorogenic acid—as well as the inhibitor oxalic acid. Molecular docking studies identified key residues involved in substrate binding, shedding light on the molecular basis of substrate specificity. Notably, the alpha-helix in the Q53-D63 region near the copper ion was extended upon substrate binding, likely stabilizing the active site and enhancing catalytic efficiency. In contrast, this helix was disrupted in the presence of the inhibitor, leading to a reduction in enzymatic activity. Furthermore, the formation of a new alpha-helix in the F350-V378 region upon substrate binding may further stabilize the substrate and contribute to improved catalytic efficiency.

This study is the first to unveil the substrate-specific catalytic mechanisms of lotus root PPO, providing theoretical support for experimentally observed differences in catalytic activity. These findings contribute to a deeper understanding of how PPO discriminates between different substrates and optimizes its catalytic function. Additionally, the research highlights the inhibitory mechanisms of oxalic acid, a potent PPO inhibitor. This study explores both the mechanisms of substrate specificity and inhibition, offering a valuable foundation for future research aimed at regulating enzymatic browning in food products through targeted PPO inhibition.

However, there are limitations to this study. The absence of an experimentally resolved crystal structure for lotus root PPO means that the protein model used contains many regions of random coils, leading to fluctuations during simulations. Achieving equilibrium required extended simulation times. Future studies that obtain and utilize a crystal structure of lotus root PPO would likely yield more accurate and detailed insights. Additionally, this study primarily focuses on three specific substrates, which, while informative, may not fully represent the broad range of phenolic compounds that PPO can interact with. Future studies should aim to obtain a high-resolution crystal structure of lotus root PPO to validate and refine our findings. Expanding the range of substrates and inhibitors studied, as well as exploring genetic modifications to PPO, could provide a more comprehensive understanding of its catalytic behavior. Furthermore, integrating experimental validation, such as enzyme kinetics and site-directed mutagenesis, would strengthen the conclusions drawn from the computational analyses and offer more actionable insights for food processing applications.

Looking forward, this research holds substantial implications for food science and technology. Understanding the catalytic mechanisms of PPO can inform the development of more effective inhibitors to prevent enzymatic browning in food products, thus improving quality and shelf-life. Additionally, this study lays the groundwork for further exploration of PPO’s role in plant physiology and its potential applications in agricultural biotechnology. Future research directions could include the investigation of PPO interactions with a broader range of substrates and inhibitors, as well as the exploration of genetic modifications to enhance desirable PPO functions or suppress undesirable activities.

In conclusion, this study advances our knowledge of PPO’s catalytic mechanisms, offering theoretical foundations for practical applications and future research avenues aimed at optimizing food quality and extending our understanding of plant biochemistry.

## 4. Materials and Methods

### 4.1. Preparation of Simulated Molecular Systems

The lotus root polyphenol oxidase sequence used in this study was obtained from the UniProt database (https://www.uniprot.org/, accessed on 16 January 2024) under sequence number E5L9E4 [33]. Protein structures were obtained using Alpha Fold 3 [34], removing the part of the tail at the end that could not be modeled. After modeling, the system was performed with equilibration simulation for 150 ns to obtain a more stable equilibrium structure for further molecular dynamics simulation. To ensure the quality of the homology-modeled structure, we conducted several validation steps. Initially, the model was assessed using the ipTM (interface predicted Template Modeling) and pTM (predicted Template Modeling) scores, which are common metrics derived from the Template Modeling (TM) score to evaluate the accuracy of predicted protein structures and their interfaces. The ipTM value of 0.98 and pTM value of 0.79 for this structure indicate that it has high structural reliability. Additionally, we performed an Expected Position Error (EPE) analysis to assess the positional accuracy of the retained residues, confirming the model’s reliability. To further strengthen the validation, we also conducted a Ramachandran Plot Analysis [35]. Using the PROCHECK [36] tool (https://www.ebi.ac.uk/thornton-srv/software/PROCHECK/), we generated a Ramachandran plot (Figure S5), which showed that the majority of the residues are located within the allowed regions (core or favored regions), verifying the geometric quality and correct distribution of the backbone dihedral angles in the model. These comprehensive validation steps indicate that the generated lotus root polyphenol oxidase model is robust and suitable for subsequent molecular dynamics simulations and other computational analyses. The three substrates selected for this study—(+)-catechin, (−)-epicatechin, and chlorogenic acid—were chosen based on their ranking in catalytic activity from a previous study. These substrates were selected to explore the enzyme’s substrate specificity and to examine the molecular interactions that contribute to the unique catalytic behavior of lotus root PPO. In addition to the substrate studies, we included oxalic acid, a known potent inhibitor of PPO identified in previous research, to examine its effects on substrate specificity and to further understand the inhibitory mechanisms of lotus root PPO. Small molecule structures were obtained from the PubChem database [37] (https://pubchem.ncbi.nlm.nih.gov/, accessed on 16 January 2024) for (+)-catechin (CID: 9064), chlorogenic acid (CID: 1794427), and (−)-epicatechin (CID: 72276). The molecular docking was carried out using Autodock Vina 1.2.0 [38,39,40] for the study. The coordinates of the center of the docking site were x = 17.000, y = 39.600, z = 71.991, and the box size was 25.00 Å × 25.00 Å × 25.00 Å. A total of four molecular dynamics simulation systems were set up for the study, namely ligand-free protein system, catechin-protein system, epicatechin-protein system, and chlorogenic acid-protein system.

### 4.2. Conventional Molecular Dynamics Simulations

AMBER 22 [41] software was used to run conventional molecular dynamics (cMD) simulations utilizing the pmemd.cuda module [42]. Force field parameters for proteins were produced using the ff19SB [43] force field in the Leap module prior to simulation. Ligands were parameterized using the General AMBER Force Field (GAFF) with atomic charges derived from the AM1-BCC method. Every system was solved using the OPC [44] water model inside an octahedral box. In order to reduce edge effects, periodic boundary conditions (PBC) were used, keeping a 15 Å buffer between the solute surface and the box edges. Na+ ions were introduced to the systems in order to neutralize them. The particle mesh Ewald (PME) approach with a cutoff of 10 Å was used to address non-bonded electrostatic interactions, while the SHAKE algorithm [45] was used to limit all connections involving hydrogen atoms. The energy minimization process consisted of 5000 steps each of the steepest descent and conjugate gradient algorithms. The systems were gradually heated to 300 K under an NVT ensemble and equilibrated for 50 ns under an NPT ensemble, employing a time step of 2 fs for the entire simulation.

### 4.3. Gaussian-Accelerated Molecular Dynamics Simulations

Equipped structures from the previous cMD simulations were used in Gaussian-accelerated molecular dynamics (GaMD) simulations [23]. Using a harmonic boost potential to lower energy barriers and smooth the potential energy surface, GaMD improves sampling and makes shifting between various conformational states easier. Dual-boost settings from an initial 50 ns cMD run were used in this investigation. Then, under an NVT ensemble, a 500 ns GaMD simulation was run, with coordinates collected every 10 ps. To ensure transparency and reproducibility, the specific parameters used in our Gaussian-Accelerated Molecular Dynamics (GaMD) simulations are detailed in the Supplementary Materials, including the full input files. These parameters were carefully chosen to optimize the accuracy and robustness of our simulations, and they include settings for the initial equilibration, production runs, and other key simulation conditions. All simulations were conducted with three independent parallel runs, with the results provided in the Supplementary Materials (Figures S6–S8, Table S1). Corresponding conformational changes were consistently observed across the parallel trajectories, confirming the reproducibility of the study.

### 4.4. Trajectory Analysis

The Cpptraj module [46,47] of AMBER 22 was utilized to calculate the trajectory analysis’s root mean square deviation (RMSD), root mean square fluctuation (RMSF), radius of gyration (R_g_), solvent-accessible surface area (SASA), dynamic cross-correlation matrices (DCCM) and distance analysis. Additionally, Cpptraj was used to perform principal component analysis (PCA) in order to record the coordinated movements of the protein [48]. To determine the main structural states and associated energy barriers, free energy landscapes (FEL) were created [49].

### 4.5. MM-PBSA Calculations

Binding free energy calculations between proteins and substrates were performed using the molecular mechanics/Poisson–Boltzmann surface area [50,51] (MM/PBSA) method. The binding free energy (ΔG_bind) was determined as follows:

ΔG_bind = ΔH − TΔS,(1)

The solvate entropy term was not determined because the changes in the protein and ligand following binding were identical in all systems, with extremely tiny entropy differences. The enthalpy change (ΔH) was calculated by adding the average changes in the solvation-free energy (ΔG_sol_) and the gas phase energy (ΔE_MM_) over a conformational ensemble produced using MD simulations:

ΔH = ΔE_MM_ + ΔG_sol_, (2)

To estimate ΔE_MM_, the following formula was applied:

ΔE_MM_ = ΔE_ele_ + ΔE_vdW_ + ΔE_int_, (3)

where ΔE_ele_, ΔE_vdW_, and ΔE_int_ stand for the electrostatic energies, vdW energies, and internal energies corresponding to the bond, angle, and dihedral energies, respectively.

The protein–ligand complex and protein and ligand conformational structures were acquired from a single MD trajectory (just the complex trajectory) in this study, which treated the protein–ligand structure as a rigid body. Because this energy term was computed from the same MD-simulated trajectory, the ΔE_int_ between the complex and the isolated components could therefore offset each other.

Moreover, in the subsequent investigation, just the ΔE_ele_ and ΔE_vdW_ of Equation (3) were examined.

The total of the polar solvation-free energy (ΔG_pb_) and non-polar solvation-free energy (ΔG_np_) was denoted by the symbol ΔG_sol_:

ΔG_sol_ = ΔG_pb_ + ΔG_np_, (4)

The linearized Poisson–Boltzmann equation was solved using the PBSA tool in the AMBER 22 suite to determine ΔG_pb_ [52,53]. To calculate MM/PBSA, 50 snapshots were taken from the final trajectory [54].

### 4.6. Markov Model Analysis

Following the AMBER tutorials, we performed a Markov model analysis using the PyEMMA 2.5.7 package [55]. We analyzed the 500 ns trajectory data for each system, focusing on reducing noise and computational costs by estimating the channel size as a feature with the MDAnalysis 2.2.0 package. We calculated the free energy barriers (ΔG) between metastable states in a Markov state model (MSM) by identifying transition pathways using Transition Path Theory (TPT) and computing the Mean First Passage Time (MFPT) for each transition. These free energy barriers offer insights into the stability and kinetics of the state transitions. The minimum RMSD was also utilized as a feature. The lag time was selected based on the relatively resolved timescales, as indicated by the implied timescales (ITS) shown in Figure S9. A Chapman–Kolmogorov test was conducted for each system, and all four systems successfully passed validation, as demonstrated in Figure S10.

## 5. Conclusions

This study employed Gaussian-accelerated molecular dynamics (GaMD) simulations to investigate the catalytic efficiency mechanisms of lotus root polyphenol oxidase (PPO) with different substrates, including catechin, epicatechin, chlorogenic acid, and the inhibitor oxalic acid. The key findings reveal significant conformational changes in PPO that correlate with its enzymatic activity. The alpha-helix in the Q53-D63 region, near the copper ion, extends upon substrate binding, likely stabilizing the active site and enhancing catalysis. In contrast, this helix is disrupted when the inhibitor binds, leading to decreased enzymatic efficiency. Additionally, the F350-V378 region, which covers the substrate-binding site, forms an alpha-helix upon substrate binding, further stabilizing the substrate and promoting catalytic function. However, in the presence of the inhibitor, this alpha-helix does not form, destabilizing the binding site and inhibiting activity. This research provides the first detailed analysis of the substrate-specific catalytic mechanisms of lotus root PPO, revealing insights that can improve food quality and shelf life.

## Figures and Tables

**Table 1:** Free energy barriers (ΔG) between metastable states calculated using transition path theory (TPT) in kcal/mol.

	S1–S2	S1–S3	S1–S4	S2–S3	S2–S4	S3–S4
Ligand-free protein	3.95	3.69	3.18	2.83	3.23	3.09
Catechin-bound protein	3.78	3.70	3.82	3.86	3.77	3.96
Epicatechin-bound protein	2.31	4.28	4.26	4.13	4.17	4.25
Chlorogenic acid-bound protein	2.96	2.62	4.36	1.78	4.33	4.25
Oxalic acid-bound protein	2.83	3.99	4.18	3.77	4.04	2.87

**Table 2:** MM-PBSA (kJ/mol) of the five systems.

	Catechin	Epicatechin	Chlorogenic Acid	Oxalic Acid
∆E_vdW_	−19.32 ± 1.17	−22.50 ± 2.10	−51.40 ± 1.17	−0.76 ± 0.44
∆E_ele_	−51.65 ± 2.97	−46.71 ± 5.13	−22.35 ± 3.00	−3.57 ± 2.53
∆G_gas_	−70.96 ± 2.83	−69.21 ± 3.91	−73.76 ± 3.82	−4.33 ± 2.94
∆G_solv_	59.43 ± 2.07	58.46 ± 2.80	45.73 ± 3.27	4.02 ± 2.62
∆G_total_	−11.53 ± 1.00	−10.75 ± 1.29	−28.03 ± 1.04	−0.31 ± 0.36

## Data Availability

Data is contained within the article and Supplementary Materials.

## Supplementary Materials

The following supporting information can be downloaded at: https://www.mdpi.com/article/10.3390/ijms251810074/s1.

## Abbreviations

The following abbreviations are used in this manuscript:

AMBER	Assisted Model Building with Energy Refinement
cMD	Conventional Molecular Dynamics
DCCM	Dynamic Cross-Correlation Matrix
DFT	Density Functional Theory
FEL	Free Energy Landscape
GaMD	Gaussian-Accelerated Molecular Dynamics
HOMO	Highest Occupied Molecular Orbital
ITS	Implied Timescales
LUMO	Lowest Unoccupied Molecular Orbital
MD	Molecular Dynamics
MFPT	Mean First Passage Time
MM/PBSA	Molecular Mechanics/Poisson–Boltzmann Surface Area
MSM	Markov State Model
NPT	Isothermal-Isobaric Ensemble (from French: Normale-Isobare)
NVT	Constant Number, Volume, and Temperature (from French: Normale-Volume-Température)
PCA	Principal Component Analysis
PME	Particle Mesh Ewald
PPO	Polyphenol Oxidase
PBC	Periodic Boundary Conditions
RMSD	Root Mean Square Deviation
RMSF	Root Mean Square Fluctuation
R_g_	Radius of Gyration
SASA	Solvent Accessible Surface Area
TPT	Transition Path Theory
ΔE_ele_	Electrostatic Energies
ΔE_MM_	Gas Phase Energy
ΔE_vdW_	Van der Waals Energies
ΔG_sol_	Solvation-Free Energy
Amino Acid Single-Letter Abbreviation	
F	Phenylalanine
G	Glycine
L	Leucine
V	Valine
N	Asparagine
Q	Glutamine
S	Serine
