# Supplementary material for: Specific Substrate Activity of Lotus Root Polyphenol Oxidase: Insights from Gaussian-Accelerated Molecular Dynamics and Markov State Models
Source: Int J Mol Sci. 2024 Sep 19;25(18):10074. doi: 10.3390/ijms251810074 (PMC11432685; doi:10.3390/ijms251810074)

Supplementary Material

Figure S1 Molecular Docking Results of Catechin
Figure S2 Molecular Docking Results of Epicatechin
Figure S3 Molecular Docking Results of Chlorogenic acid
Figure S4 Molecular Docking Results of Oxalic acid
Figure S5 Ramachandran Plot of PPO
Specific Parameters for cMD During Pre-equilibration
Specific Parameters for GaMD
Figure S6 RMSD Results of Parallel Trajectories
Figure S7 R_g Results of Parallel Trajectories
Figure S8 SASA Results of Parallel Trajectories
Table S1 Probability of α-Helix Formation in Residue Ranges D53-Q63 and F350-V378 Across Five Systems with Three Parallel Trajectories
Figure S9 Implied Timescales (ITS) analysis results
Figure S10 Chapman-Kolmogorov test results

Figure S1 Molecular Docking Results of Catechin

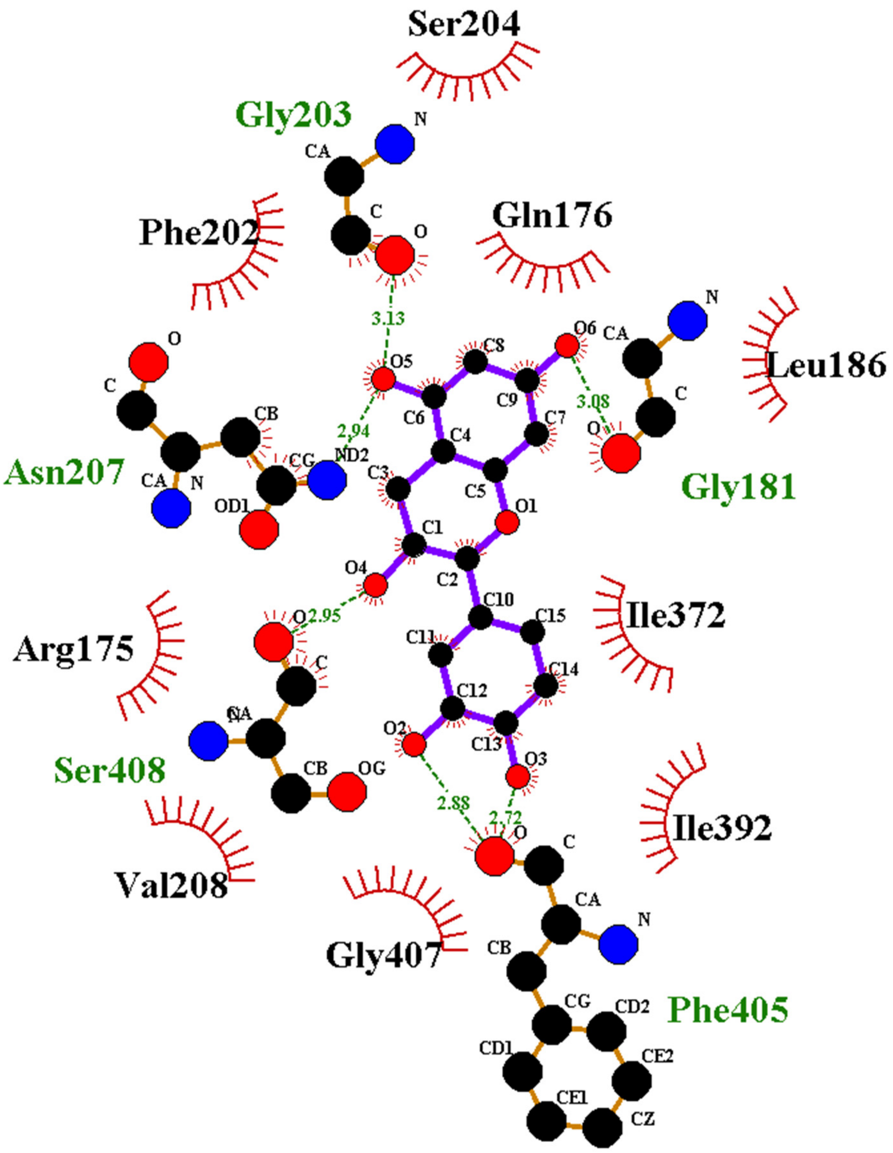

Figure S2 Molecular Docking Results of Epicatechin

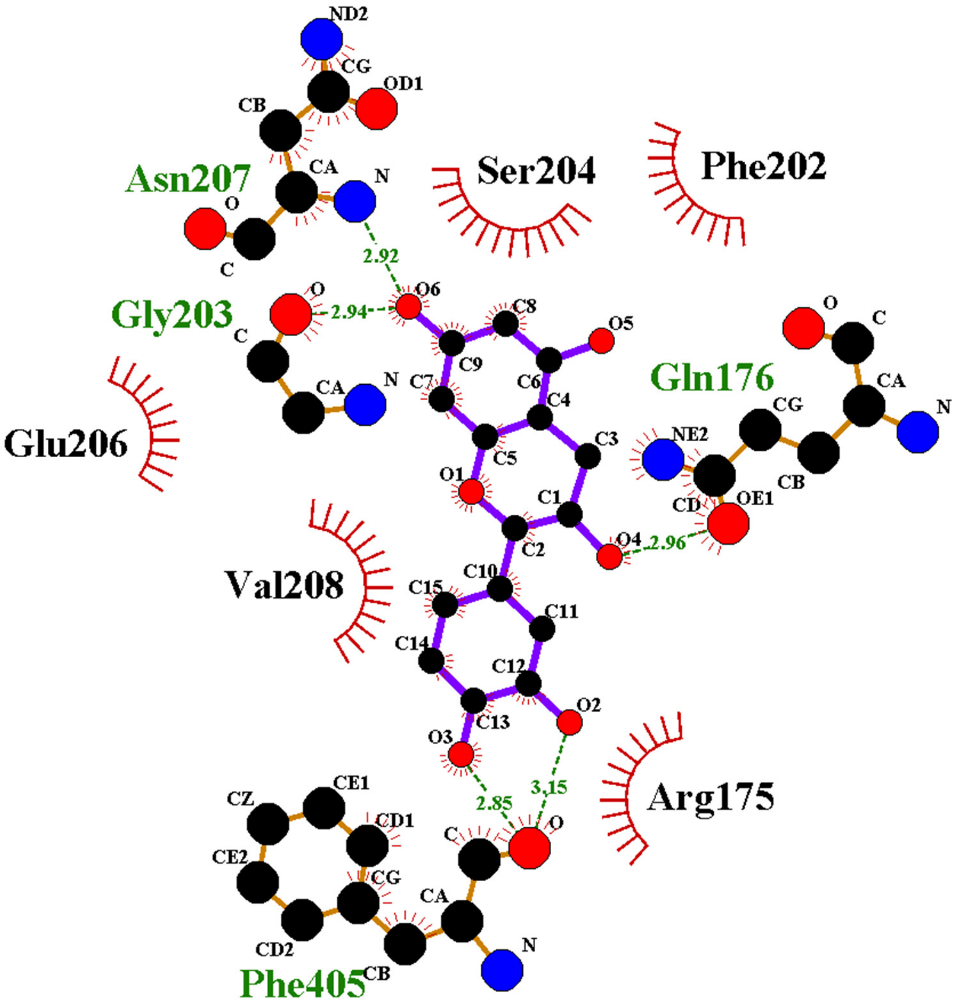

Figure S3 Molecular Docking Results of Chlorogenic acid

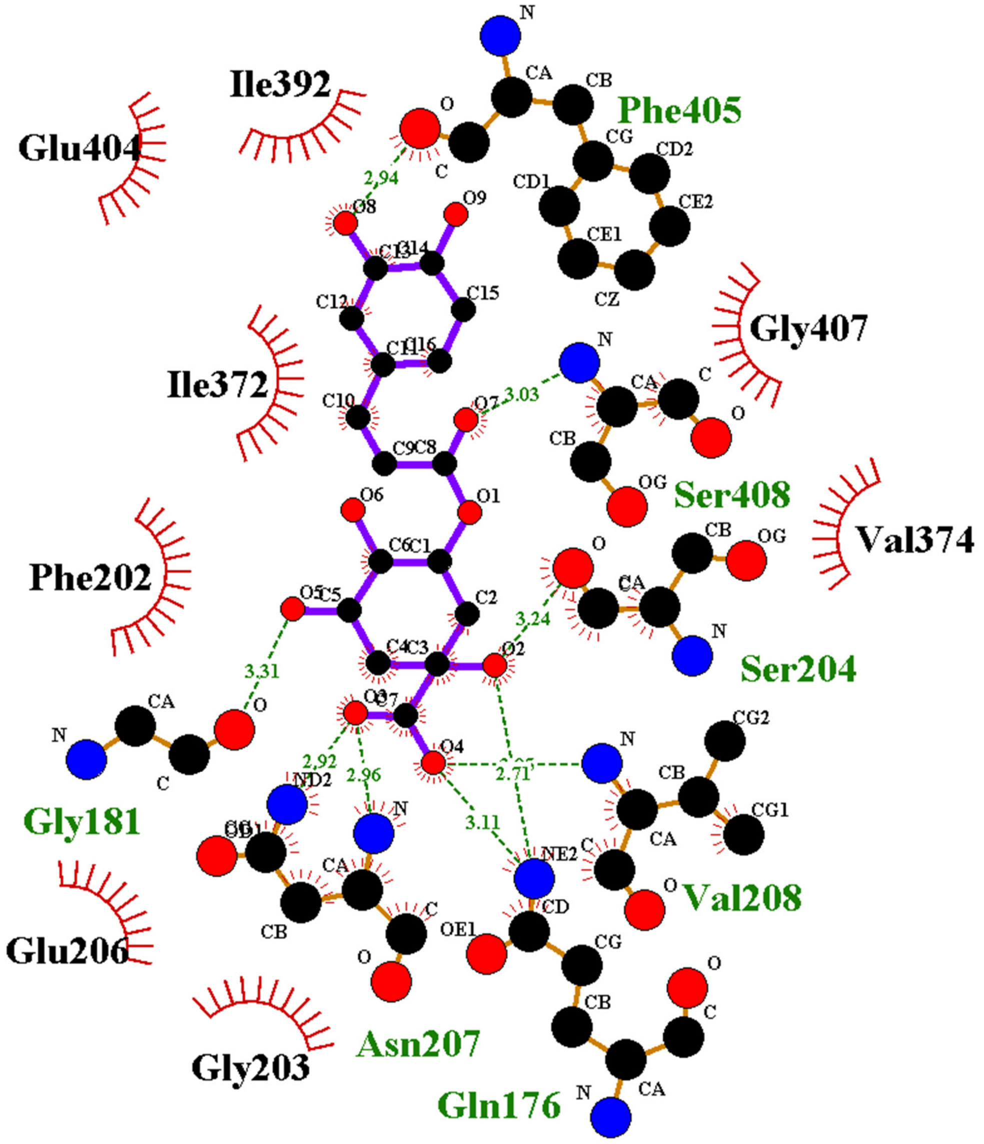

Figure S4 Molecular Docking Results of Oxalic acid

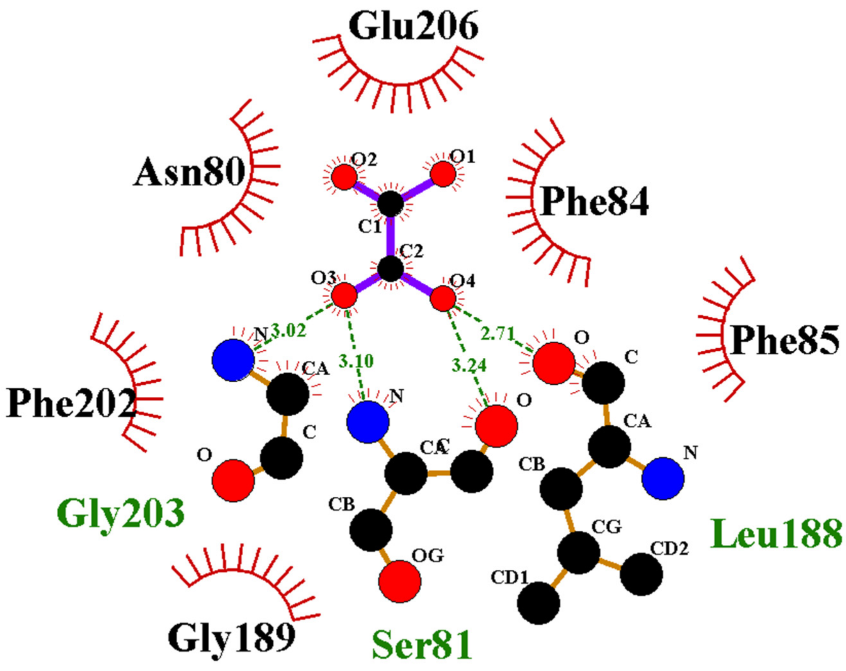

Figure S5 Ramachandran Plot of PPO

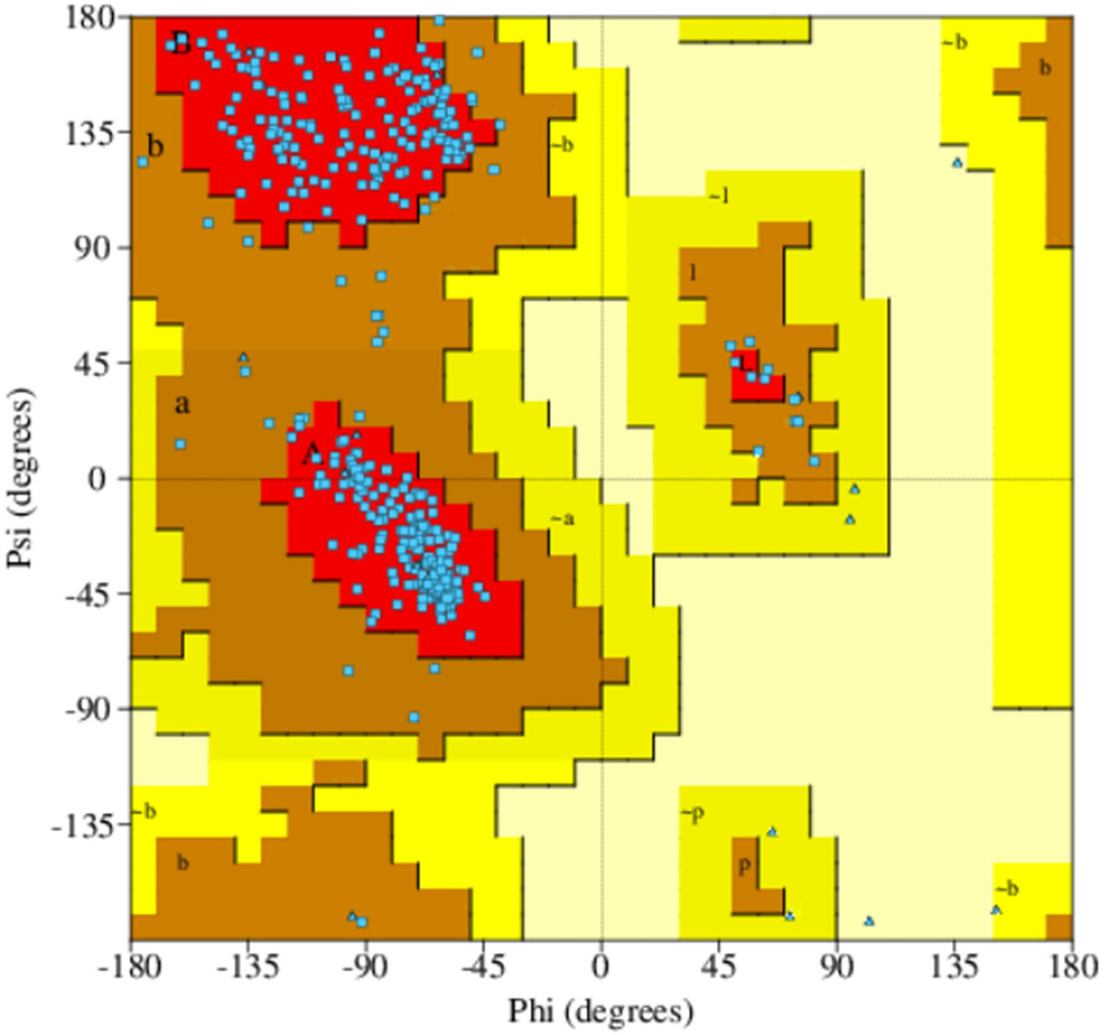

Specific Parameters for cMD During Pre-equilibration

```
&cntrl
   imin = 0,
   irest = 0,
   ntx = 1,
   ioutfm = 1,
   nstlim = 25000000,
   dt = 0.002,
   ntt = 3,
   gamma_ln = 5.0,
   ig = -1,
   tempi = 300.0,
   temp0 = 300.0,
   ntp = 0,
   ntb = 1,
   ntc = 2,
   ntf = 2,
   cut = 8,
   ntwr = 50000,
   ntpr = 5000,
   ntwx = 50000,
   ntwe = 50000,
   iwrap = 1,
   ntr = 0,

   igamd = 3, iE = 1, irest_gamd = 0,
   ntcmd = 1000000, nteb = 25000000, ntave = 200000,
   ntcmdprep = 200000, ntebprep = 800000,
   sigma0P = 6.0, sigma0D = 6.0,
/
```

Specific Parameters for GaMD

```
&cntrl
   imin = 0,
   irest = 1,
   ntx = 5,
   ioutfm = 1,
   nstlim = 250000000,
   dt = 0.002,
   ntt = 3,
   gamma_ln = 2.0,
   ig = -1,
   tempi = 300.0,
   temp0 = 300.0,
   ntp = 0,
   ntb = 1,
   ntc = 2,
   ntf = 2,
   cut = 8,
   ntwr = 50000,
   ntpr = 5000,
   ntwx = 50000,
   ntwe = 50000,
   iwrap = 1,
   ntr = 0,

   igamd = 3, iE = 1, irest_gamd = 1,
   ntcmd = 0, nteb = 0, ntave = 200000,
   ntcmdprep = 0, ntebprep = 0,
   sigma0P = 6.0, sigma0D = 6.0,
/
```

Figure S6 RMSD Results of Parallel Trajectories

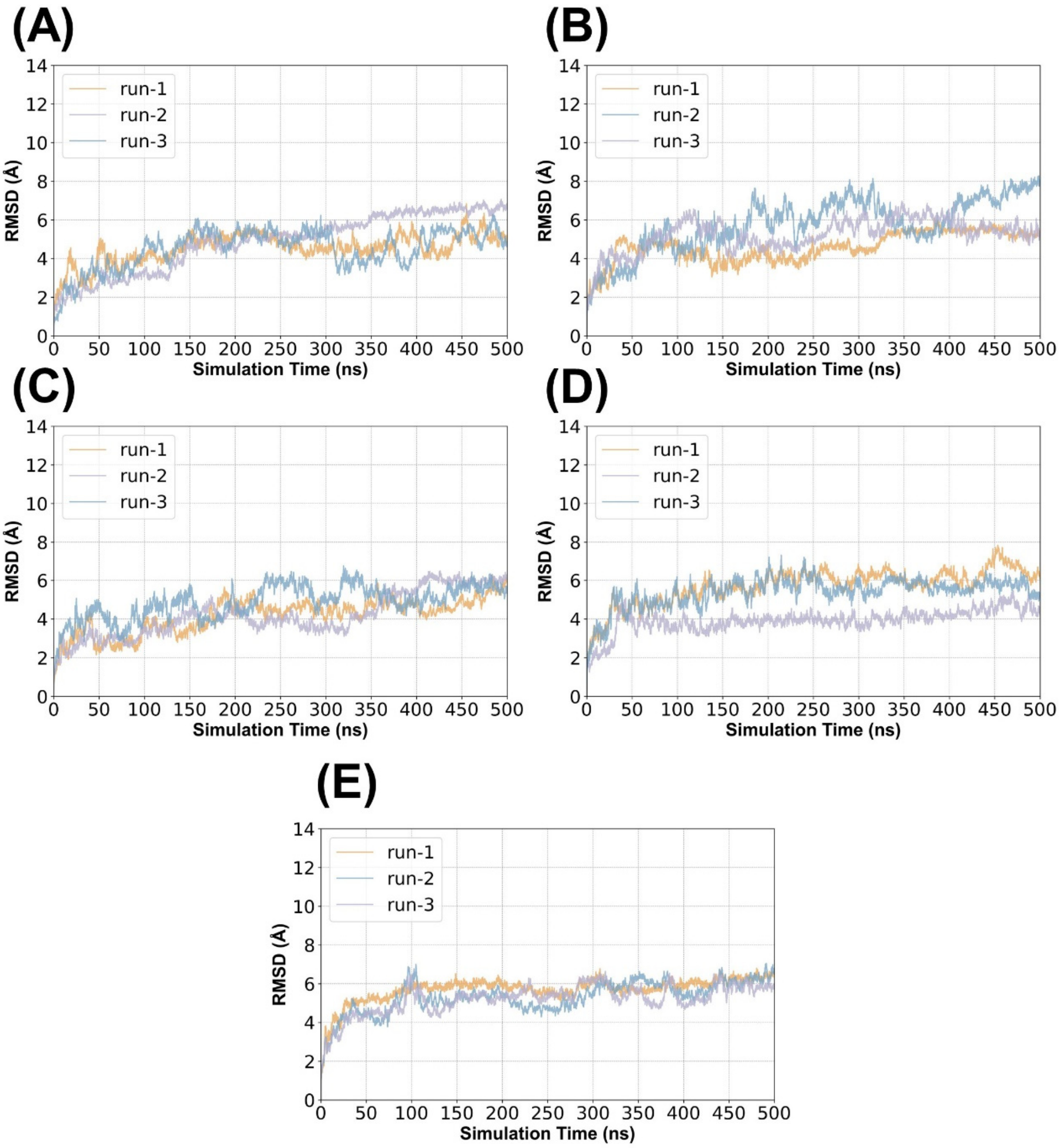

Figure S7 R_g Results of Parallel Trajectories

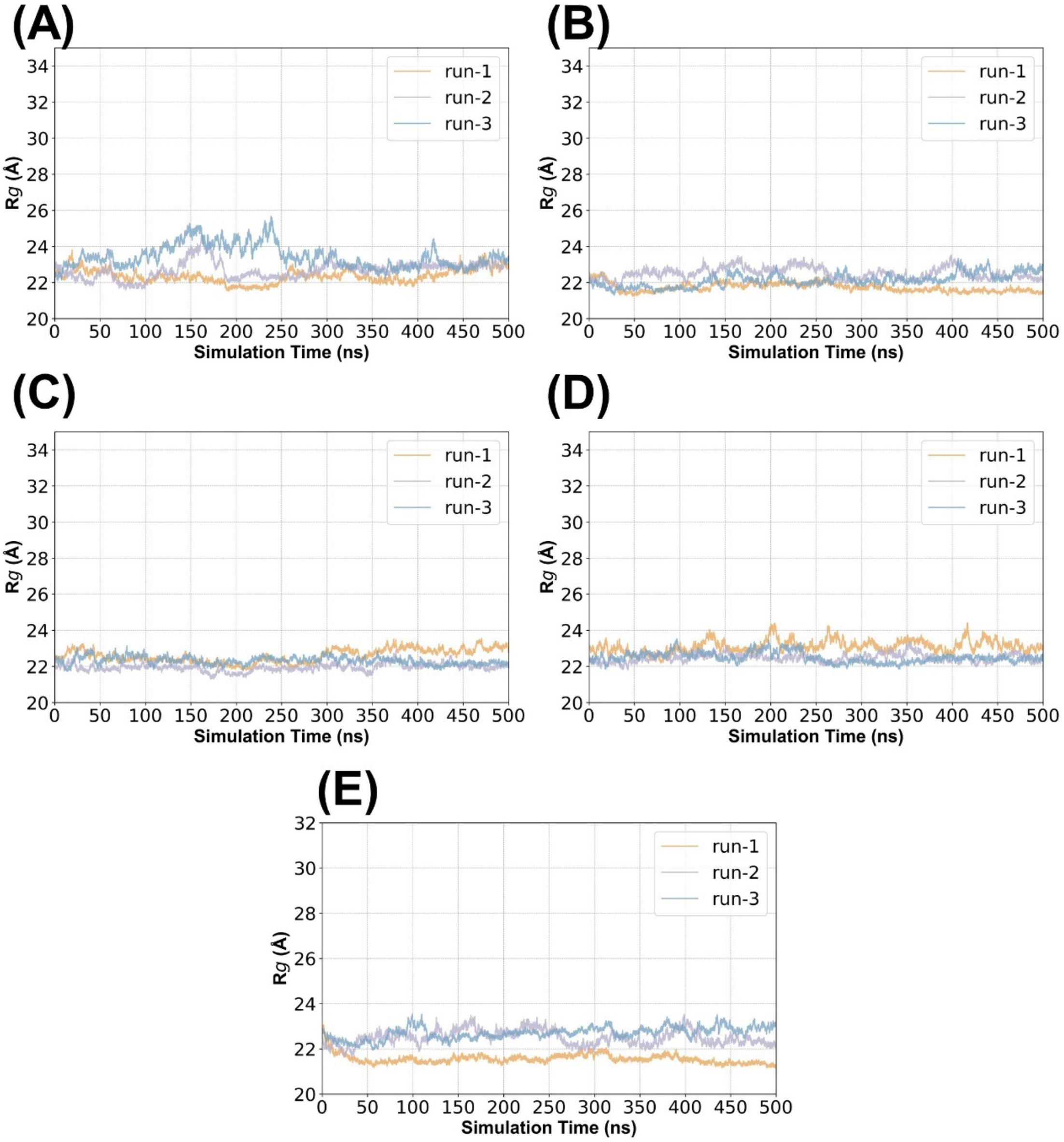

Figure S8 SASA Results of Parallel Trajectories

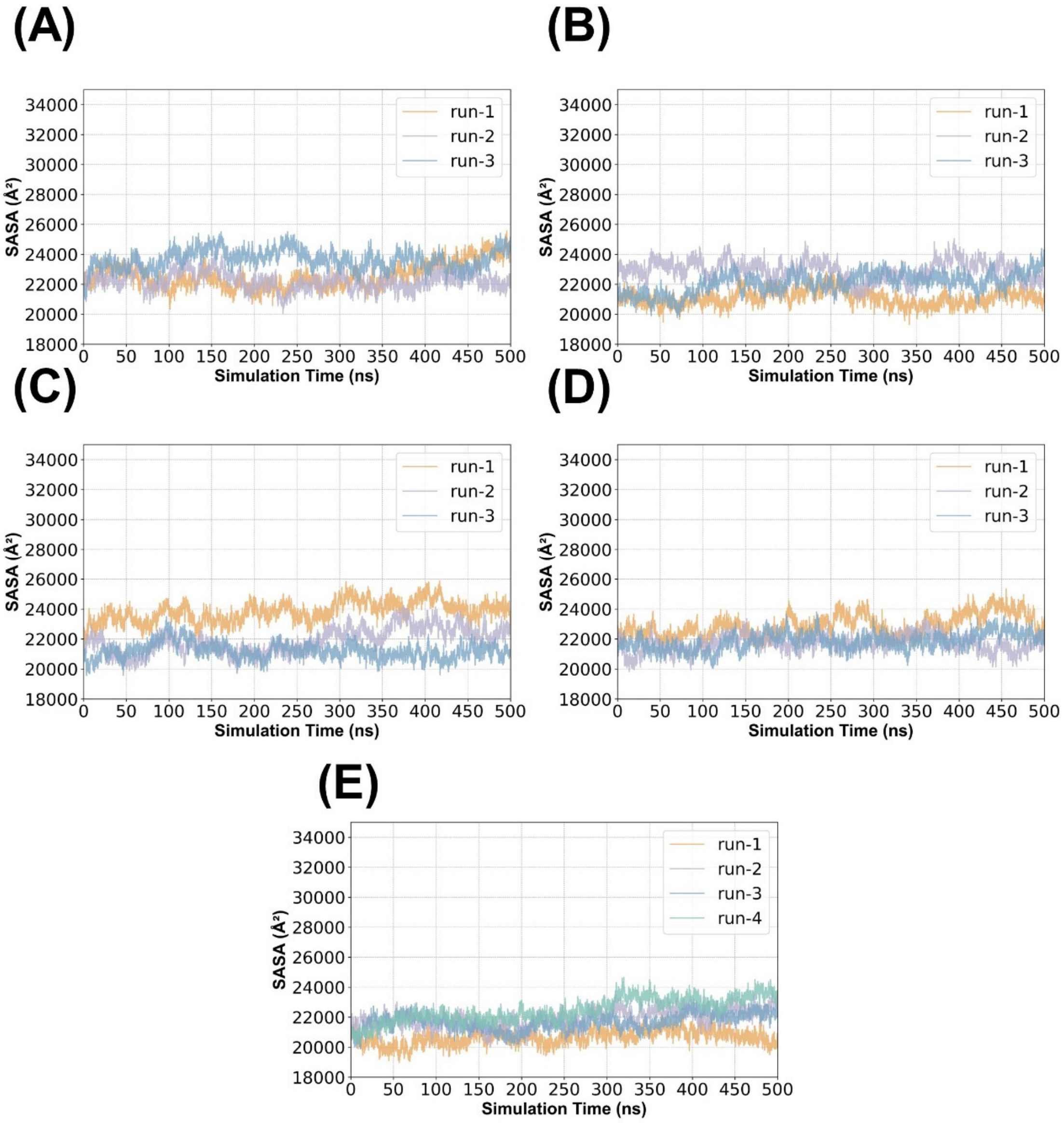

Table S1 Probability of α-Helix Formation in Residue Ranges D53-Q63 and F350-V378 Across Five Systems with Three Parallel Trajectories

System	D53-Q63	F350-V378
Ligand-free protein(run-1)	18.82%	3.08%
Ligand-free protein(run-2)	20.46%	5.47%
Ligand-free protein(run-3)	17.38%	3.72%
Ligand-free protein(Average)	18.89%	4.09%
Catechin-bounded protein(run-1)	36.51%	12.82%
Catechin-bounded protein(run-2)	37.75%	11.76%
Catechin-bounded protein(run-3)	29.48%	17.16%
Catechin-bounded protein(Average)	34.58%	13.91%
Epicatechin-bounded protein(run-1)	65.88%	19.71%
Epicatechin-bounded protein(run-2)	57.79%	16.34%
Epicatechin-bounded protein(run-3)	60.43%	20.18%
Epicatechin-bounded protein(Average)	61.37%	18.74%
Chlorogenic acid-bounded protein(run-1)	24.99%	4.79%
Chlorogenic acid-bounded protein(run-2)	27.31%	4.35%
Chlorogenic acid-bounded protein(run-3)	21.07%	6.14%
Chlorogenic acid-bounded protein(Average)	24.46%	5.09%
Oxalic acid-bounded protein(run-1)	22.42%	2.71%
Oxalic acid-bounded protein(run-2)	25.61%	4.48%
Oxalic acid-bounded protein(run-3)	20.87%	2.59%
Oxalic acid-bounded protein(Average)	22.97%	4.16%

Figure S9 Implied Timescales (ITS) analysis results

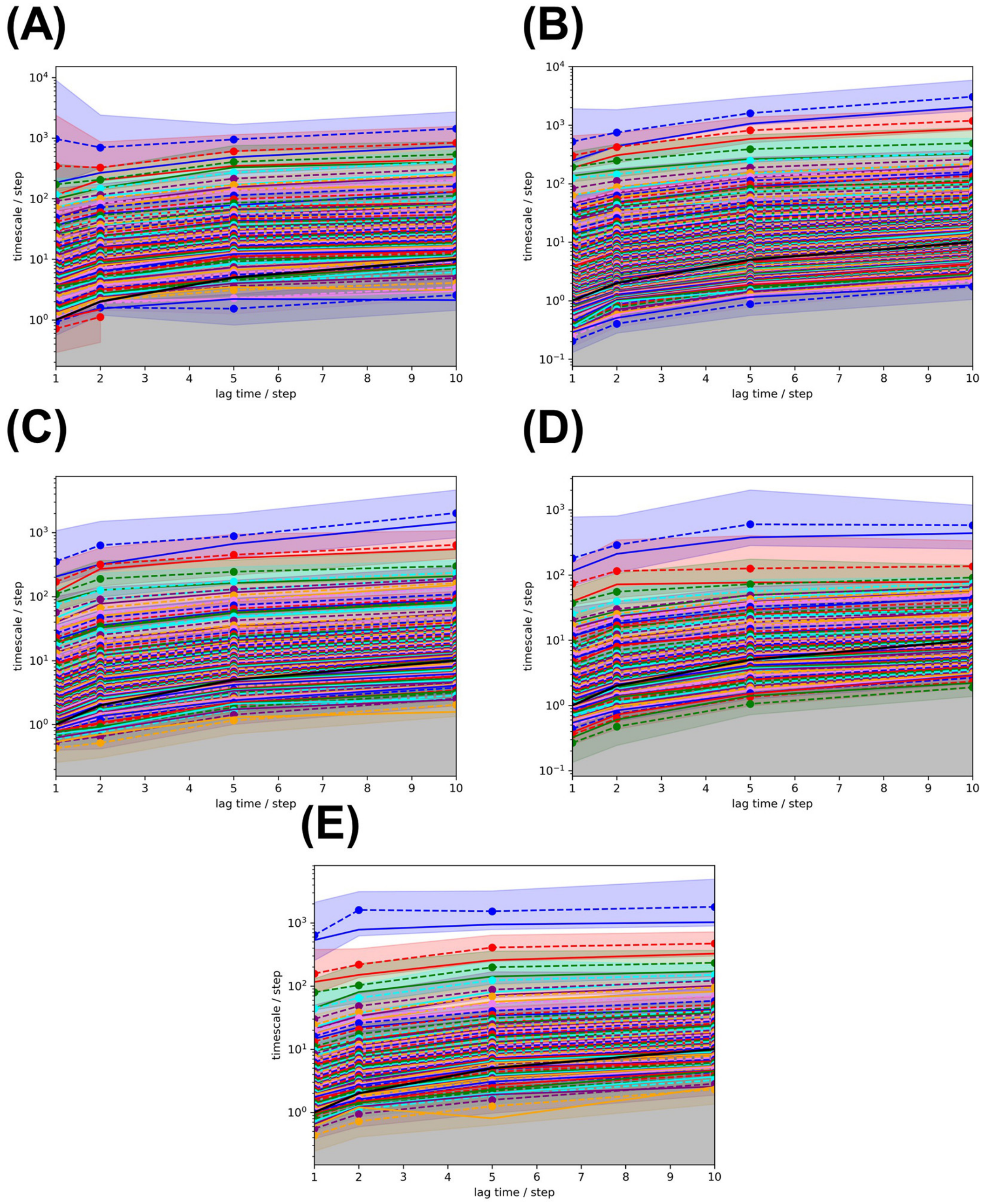

Figure S10 Chapman-Kolmogorov test results

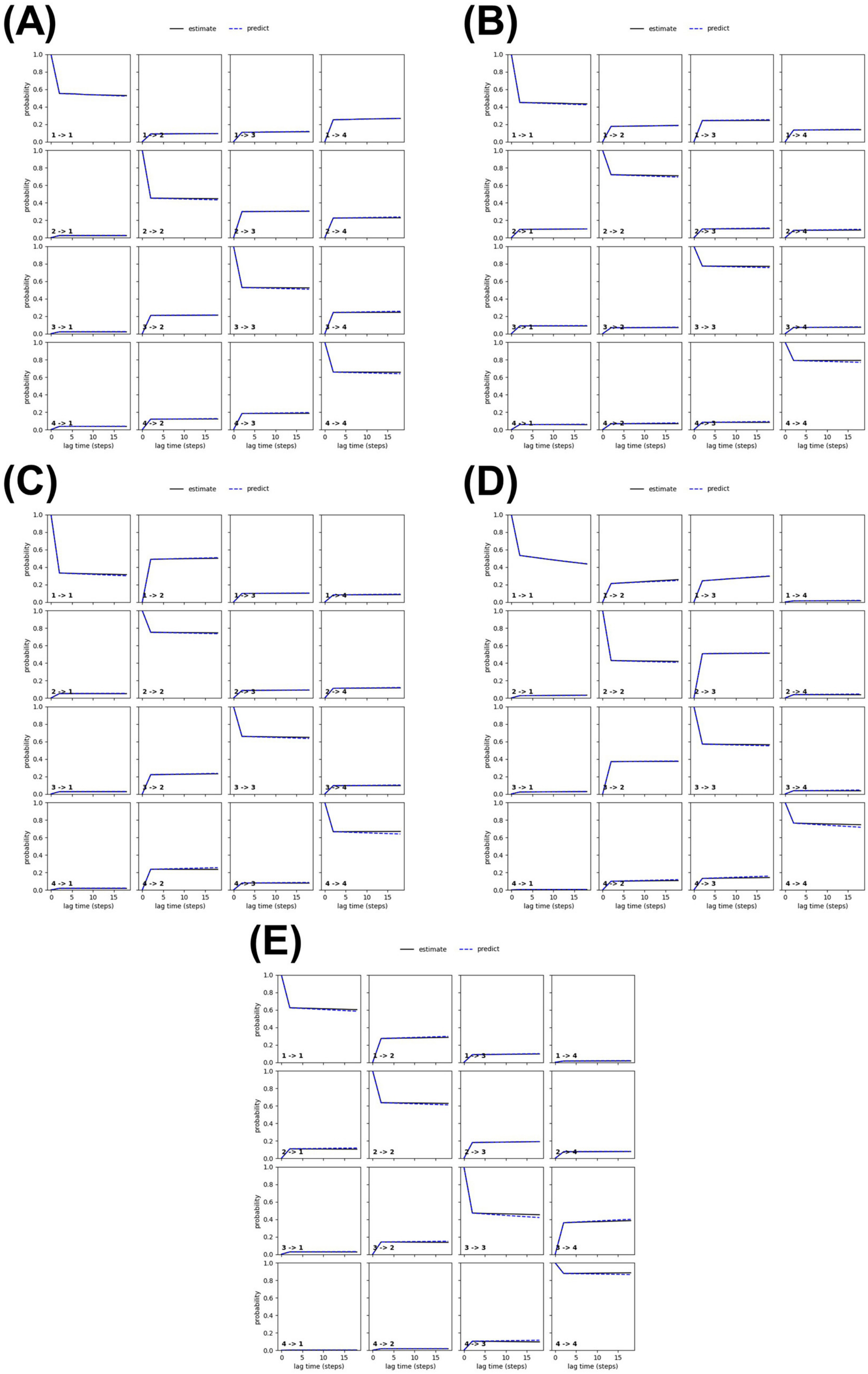

Supplement: Supplementary file 1 [file ijms-25-10074-s001.zip › ijms-3143223-supplementary.pdf]
